# Inferior vena cava diameter measurements and BUN/creatinine values to determine dehydration in patients with hip fractures preoperatively: A prospective observational study

**DOI:** 10.1097/MD.0000000000015197

**Published:** 2019-04-26

**Authors:** Ayhan Kaydu, Erhan Gokcek

**Affiliations:** Department of Anesthesiology, Diyarbakir State Hopital, Turkey.

**Keywords:** BUN/creatinine ratio, collabsibility index, dehydration, hip fracture, inferior vena cava diameter

## Abstract

Dehydration is a common problem in patients undergoing hip fracture surgery. Sonographic inferior vena cava (IVC) diameter measurement evaluates to estimate volume status. The aim of the study to evaluate the relationship between IVC measurements (expiratory diameter of IVC, collabsibility index [CI], inspiratory diameter of IVC) and blood urea nitrogen (BUN)/creatinine ratio in patients undergoing hip fracture surgery. Ultrasonography of IVC was performed on 35 patients underwent hip fracture surgery. The end-expiratory diameter of IVC, end-inspiratory diameter of IVC, and CI were assessed preoperatively. The patients were classified as group 1 for BUN/Cr ratio <20, group 2 for BUN/Cr ratio of >20. Sonographic IVC measurement was not successful in 14.2% of patients and 30 patients remained. The mean age was 80.43 ± 11.10 (58–95) years. The IVC diameter values had no discriminatory value for the prediction of dehydration according to BUN/creatinine ratio (*P* > .05). Receiver operating characteristic curve indicated that area under the curve (AUC) for CI: 49.5%, (95% CI 26.5–72.5) *P* > .05; for IVC inspiratory diameter: AUC: 43.3%, (95% CI, 19.9–66.6) *P* > .05; for IVC expiratory diameter: AUC: 45.5%, (95% CI, 26.6–65.4) *P* > .05. No correlations of BUN/creatinine ratio with CI and IVC expiratory diameter were found (as r = −0.262 [*P* = .163]; [*r* = 0.206, *P* = .274]; respectively). There were not any correlation in linear regression analysis model between BUN/Cr ratio according to independent variables (Age, CI, IVCmax, IVCmin) (*P* = .108, *P* = .419, *P* = .282, *P* = .257; respectively). No discriminatory relationship was found between the bedside ultrasonographic measurement of IVC parameters and BUN/creatinine ratio in patients underwent hip fracture surgery to predict the preoperative dehydration.

## 1 Introduction

Hip fractures or proximal femoral fractures (PFF) are common and serious injuries that lead to high morbidity and mortality rates, as well as significant economic burdens around the world.^[1]^ Due to the increase in the elderly population, the expectation is that the number of hip fractures will be on the rise in the coming years. Surgical treatment is the first-line method for most PFF and preoperative comorbidities are common among older patients besides dehydration due to the loss of both total body fluids and blood loss which is frequent on arrival to the hospital.^[2]^

Dehydration is defined as “a complex condition resulting in a reduction in total body water.”^[3]^ The reasons of dehydration can be caused by water loss or salt loss dehydration. These 2 types of dehydration are seen in elderly patients with hip fractures due to both inadequate fluid intake and blood loss. Moreover, the decrease of the feeling of thirst, intake of some fluid losing drugs (eg, diuretics), and loss of physiologic functions contribute to dehydration in this patient group. Although many different methods can be used to determine dehydration, none is the gold standard.^[4]^ The plasma blood urea nitrogen (BUN)/creatinine (Cr) ratio is a test frequently used and cited in the literature for the definition of dehydration.^[5]^

To assess the dehydration and volume status of a patient is a big challenge for clinicians. Recently, bedside ultrasonography (USG) has been popular as being a cost-effective, rapid and noninvasive means despite some limitations. The inferior vena cava (IVC) diameter measurements variations due to respiration have become an important method for determining volume status and fluid responsiveness.^[6,7]^ In addition, different studies have examined the BUN/Cr ratio and IVC diameter measurements in elderly patients for the diagnosis of dehydration.^[8,9]^

The aim of this study was to define the relationship between sonographic IVC measurements (collapsibility index [CI], inspiratory diameter of IVC, expiratory diameter of IVC) and BUN/Cr ratio in patients undergoing proximal femoral fracture surgery.

## 2 Materials and methods

### 2.1 Patients

This study was designed as a prospective, observational research. The study was approved by the Institutional Ethics Committee (decision no. 2017/102, Diyarbakir Training and Research Hospital Ethics Committee). The study was performed according to the Helsinki Declaration of 1975, as revised in 2000. All patients were informed and written consent obtained from all patients. The inclusion criteria for the study were patients diagnosed with PFF, patients aged over 60 years and patients who understood the study protocol and informed consent. Patients with gastrointestinal tract abnormality due to previous gastrointestinal surgery (hiatal hernia, oesophageal, hepatic, gastric surgery), increased intraabdominal pressure (eg, past abdominal surgeries), right ventricular dysfunction, major peripheral vascular disease, obvious valvular heart disease, increased pulmonary arterial hypertension and obvious right heart dysfunction, skin infection over abdominal area were excluded from the study.

### 2.2 Preoperative measurements

Either an 18G or 20G intravenous catheter was inserted in all patients. Noninvasive blood pressure, peripheral oxygen saturation and electrocardiogram were monitored preoperatively. Patients received no premedication before arrival in the operating theatre.

In the preoperative care unit, an experienced physician having IVC measurement experience with at least 50 patients in the last 6 months, performed both gastric examinations and IVC measurements of patients who were lying supine. The physician performing the USG procedure was unaware of the patient's general condition, blood values and the method of anesthesia to be administered. The USG measurement was performed with a 2 to 5 MHz curve array low-frequency transducer (Sonosite M-Turbo Bothell, WA). The results were digitally recorded. The IVC was observed by placing the transducer along the subxiphoid longitudinal axis. After identifying the conjunction of the right atrium with the IVC as a 2-dimensional image, a pulsed wave doppler was used to separate the IVC from the aorta. A time-motion record of the IVC diameter was measured in the 2-dimensional mode with M-mode imaging at 2 to 3 cm distal from the entrance of the right atrium. The highest and lowest diameters of IVC measurements at the end of both inspiration and expiration were recorded.

The IVC-CI was calculated as (inferior vena cava diameter [dIVC]-CI) = (dIVCmax − dIVCmin)/dIVCmax and expressed as a percentage (%). All scanning procedures were completed within 10 minutes.

### 2.3 Data collection

The demographic data (age, gender, height, weight), body mass index (BMI) (calculated according to BMI = weight/height^2^ formula), comorbidities, preoperative IVC values (CI, dIVCmax, dIVCmin diameter at expiration and inspiration), plasma parameters (BUN/Cr ratio), and preoperative hemodynamic values were recorded. According to the study by Riccardi et al, we determined that an extremely high degree of dehydration above the cut-off value of 20 for BUN/Cr ratio.^[9]^ Patients were classified as group 1 for BUN/Cr ratio <20 and group 2 for BUN/Cr ratio of >20.

### 2.4 Statistical analysis

Data were summarised using means and standard deviations and as percentages for discrete variables. Demographic data, laboratory values, and IVC diameters were calculated by descriptive statistics. To determine continuous variables for abnormal distribution, the Shapiro–Wilk test was used. If they were normally distributed, the central tendency was expressed as the mean (SD). Continuous variables were compared using the Mann–Whitney *U* test. A Spearman correlation analysis was used to determine the significant difference between BUN/Cr ratio and CI and IVC expiratory diameter. The receiver operating characteristic (ROC) analysis and an area under the curve (AUC) were generated to examine the performance of the tests used for the cut-off value of 20 for the BUN/Cr ratio. Multivariate regression analysis was used to predict BUN/Cr ratio according to independent variables (Age, CI, IVCmax, IVCmin). Differences were considered significant if *P* < .05. SPSS 22 (Chicago, IL) was used for statistical analysis.

## 3 Results

**Figure 1:**
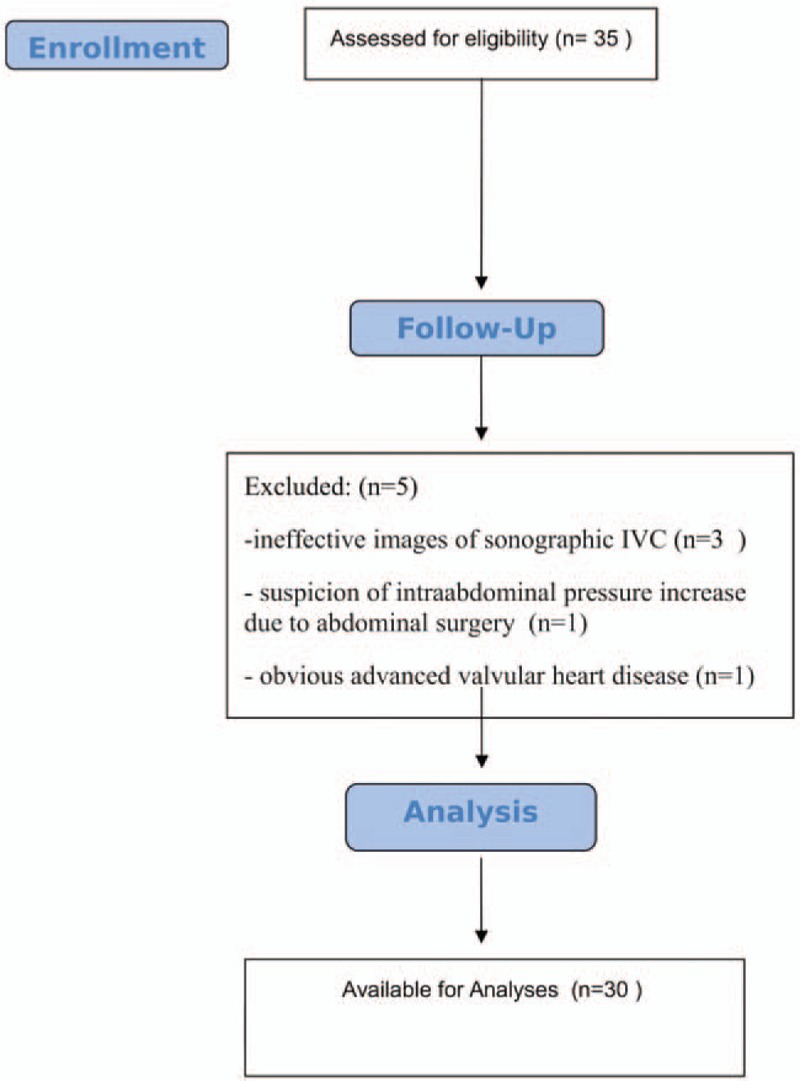
Flow diagram.

Thirty-five patients were recruited into this study. The sonographic measurement of IVC was unsuccessful in 14.2% patients (5 patients): 1 patient with suspicion of increased intra-abdominal pressure due to past abdominal surgery, 1 patient with obvious advanced valvular heart disease, and 3 patients with ineffective images of the IVC. These patients were excluded from the study. For this reason, the study was completed with 30 patients (Fig. 1).

The mean age of our patients was 80.43 ± 11.10 (58–95) years. Nine patients were male, and 21 patients were female. The most frequent comorbidities in patients were hypertension (40%) and cardiovascular disease (Table 1).

**Table 1:**
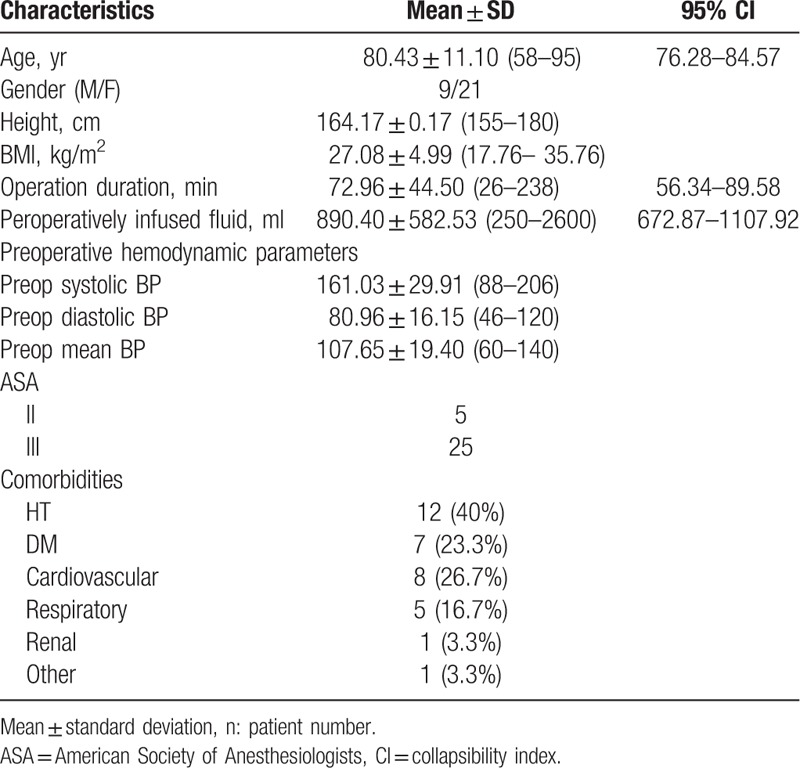
Demographic data of the patients.

The mean BUN/Cr ratio of patients was 26.44 ± 9.43 (11.67–53.98). In the dehydration group, 33.37% of patients were in the group <20 (Group I), and 66.67% were in the BUN/Cr >20 (Group II). Comparisons of groups according to their characterization are shown in Table 2. There were no significant differences in the CI and both inspiratory and expiratory diameters of IVC between groups (*P* > .05).

**Table 2:**
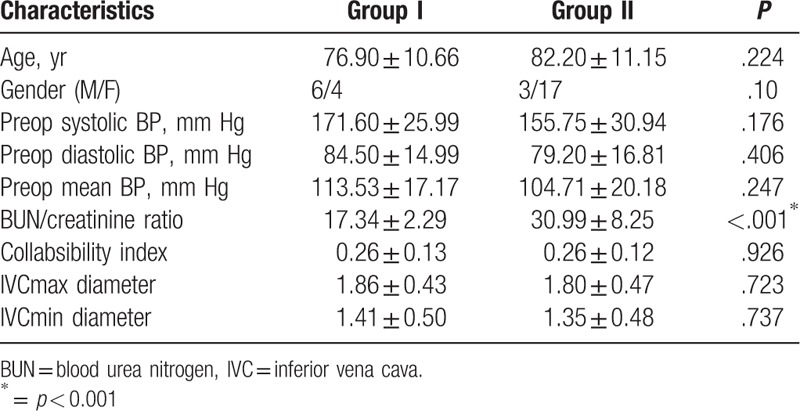
Comparison of the 2 groups divided for dehydration. (Group 1: BUN/creatinine ratio <20; Group 2: BUN/creatinine >20).

Table 3 shows the results of a linear regression analysis to predict BUN/Cr ratio according to independent variables (Age, CI, IVCmax, IVCmin). There was not any correlation between BUN/Cr ratio according to independent variables (*P* = .108, *P* = .419, *P* = .282, *P* = .257; respectively).

**Table 3:**
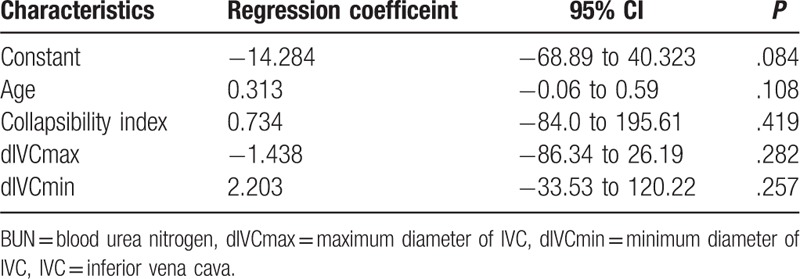
Multivariate linear regression model patients to predict BUN/creatinine ratio according to independent variables (age, CI, IVCmax, IVCmin).

**Figure 2:**
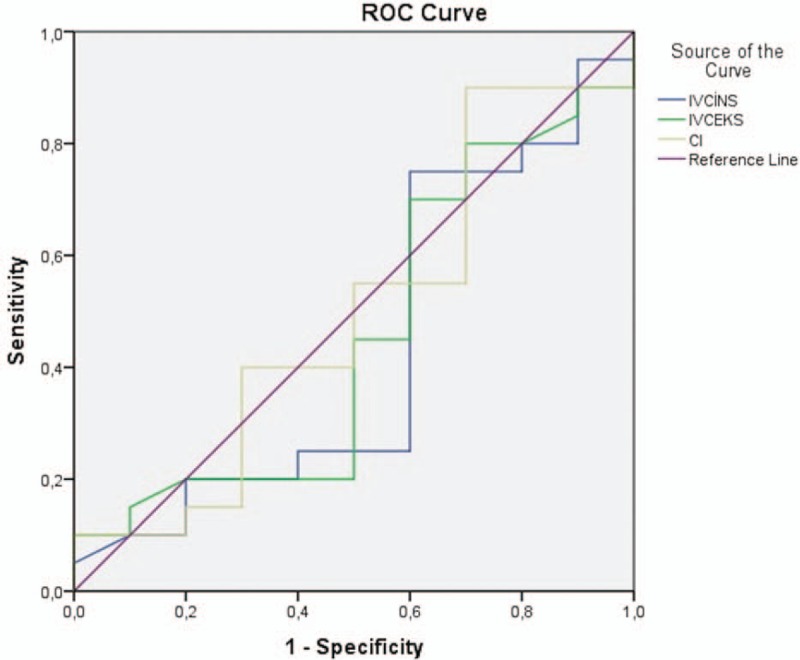
ROC curve for the collapsibility index, inspiratory, and expiratory IVC diameters according to the BUN/creatinine ratio. AUC for collapsibility index: 49.5%, (95% CI 26.5–72.5) *P* > .05; AUC for IVC inspiratory diameter: AUC: 43.3%, (95% CI, 19.9–66.6) *P* > .05; AUC for IVC expiratory diameter: AUC: 45.5%, (95% CI, 26.6–65.4) *P* > .05. AUC = area under the curve, BUN = blood urea nitrogen, CI = collapsibility index, IVC = inferior vena cava, ROC = receiver operating characteristic.

Figure 2 shows the ROC curve for the CI, inspiratory, and expiratory IVC diameters according to the BUN/Cr ratio. The IVC diameter values had no discriminatory value for the prediction of dehydration according to the BUN/Cr ratio (*P* > .05). The ROC curve indicated that the AUC for the CI: 49.5%, (95% CI 26.5–72.5) *P* > .05; for the IVC inspiratory diameter: AUC: 43.3%, (95% CI, 19.9–66.6) *P* > .05; and for the IVC expiratory diameter: AUC: 45.5%, (95% CI, 26.6–65.4) *P* > .05.

**Figure 3:**
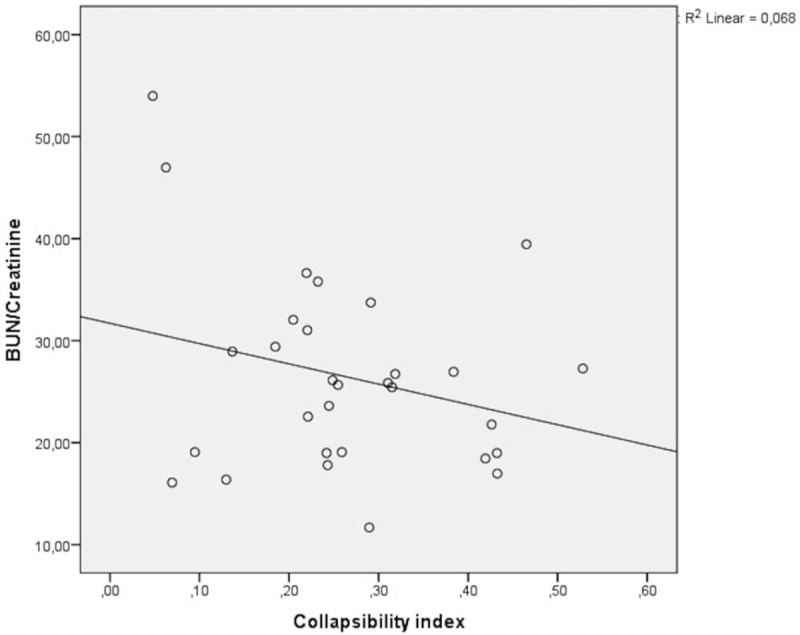
The correlation of BUN/creatinine ratio with CI. *r* = −0.262 (*P* = .163). BUN = blood urea nitrogen, CI = collapsibility index.

**Figure 4:**
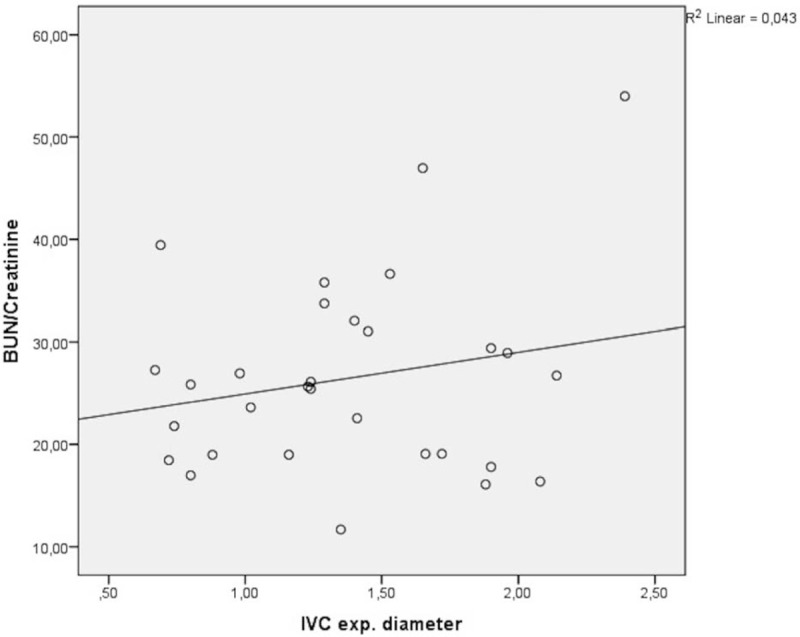
The correlation of BUN/creatinine ratio with IVC expiratory diameter. (*r* = 0.206, *P* = .274). BUN = blood urea nitrogen, IVC = inferior vena cava.

Figure 3 shows the correlation of BUN/Cr ratio with CI. There was no significant difference between BUN/Cr ratio and CI. The correlation coefficient was determined as *r* = −0.262, *P* = .163. Similar results were obtained in the relationship between BUN/Cr ratio and IVC expiratory diameter (*r* = 0.206, *P* = .274) (Fig. 4).

## 4 Discussion

In this study, we aimed to investigate the efficacy of IVC diameter measurements in determining the dehydration status of patients who underwent PFF surgery. We found that IVC diameter values (CI, inspiration and expiration diameters) did not have a discriminatory role in determining dehydration.

Hip fracture, consistent with our results, is frequently seen in older women around 80 years of age.^[10]^ Hemorrhagic bleeding by fracture, starvation, and immobility before admission to the hospital, chronic volume depletion due to decreased fluid intake in older people, comorbidities (eg, cardiovascular disease, chronic renal failure) and use of diuretics increase the sensitivity to dehydration. Occult dehydration may lead to poor tissue perfusion and overt organ failure. For this reason, both the recognition of dehydration in critically ill patients, like hip fracture and the optimization of fluid resuscitation are important issues.

Dehydration describes the loss of fluid from the intracellular compartment, unlike volume depletion, which accounts for the loss of fluid from the extracellular compartment. Different methods (bioelectrical impedance, change in total body weight, determination of total body fluid with deuterium oxide dilution, changes in serum osmolality, serum sodium levels, and plasma tonicity, etc) have been used to diagnose dehydration. In this study, we used the BUN/Cr ratio as a surrogate marker for a number of reasons: it was consistently used in both healthy and critically ill patients in different studies because it is available in every hospital worldwide, produces rapid results and is reasonably priced.^[8,9,11]^

Sonographic examination of the IVC diameter has been conducted with increased interest in predicting intravascular volume in patients with spontaneous respiration. Dynamic indices have not been shown to be effective in determining the volume status of patients with spontaneous respiration.^[12]^ Moreover, static preload indices such as central venous pressure (CVP), pulmonary arterial occlusion pressure, and systolic pressure have low predictive value.^[13]^ The USG of IVC provides reliable information to clinicians with features such as easy application and requires minimal skill to perform the procedure.^[14]^ However, USG has some technical and practical limitations such as limited imaging in patients who underwent abdominal surgery, lack of a defined uniform technique, need for new equipment, and few experienced sonographers.^[15,16]^

Two studies in the literature examined the relationship between IVC diameter and BUN/Cr ratio in elderly patients.^[8,9]^ Riccardi et al examined the relationship between BUN/Cr ratio and CI of sonographic IVC measurements in 134 patients with an average age of 62 years admitted to emergency services. They found a statistically significant correlation between CI and BUN/Cr ratio with a 60.7% (sensitivity 79% and specificity 89%) in ROC analyses (*P* < .0001). They concluded in the study that CI was a good predictor of dehydration in patients with a BUN/Cr ratio >20.^[9]^ Orso et al observed a significant correlation between CI and expiratory IVC diameter in 270 patients over 70 years of age with a BUN/Cr ratio >20 (AUC; 76% [95% CI 70–82] and 80% [95% CI 75–86]).^[8]^ In our study, we did not find any significant correlation between IVC diameter values and the BUN/Cr ratio. In contrast to the results of these studies, our study showed that the relationship between IVC diameter values and the BUN/Cr ratio had no discriminatory value for predicting dehydration.

The results of this study may have different outcomes from other studies for different reasons. The first reason is the poor predictive value of IVC measurements to predict volume status in spontaneously breathing patients, despite the demonstration of a good correlation between CVP and sonographic IVC measurements when evaluating fluid responsiveness.^[17,18]^ Since the IVC diameter is a dynamic parameter, it has been shown to be ineffective in predicting fluid responsiveness in some studies.^[12]^ Muller and colleagues reported that high IVC-CI values (>40%) were associated with fluid responsiveness in spontaneously breathing patients with acute respiratory failure, whereas low IVC-CI values (<40%) values did not exclude fluid response to volume expansion.^[19]^ Corl et al recently showed that in adult emergency department patients, the IVC-CI did not have utility to predict fluid responsiveness.^[20]^ Also, Sobzcky et al reported similar results that bedside sonographic measurements of IVC parameters did not predict fluid responsiveness after elective coronary artery bypass grafting in mechanically ventilated patients.^[21]^ Moreover, a meta-analysis including 17 prospective studies showed that respiratory variations in IVC diameter parameters in spontaneously breathing patients had limited value to predict fluid responsiveness. ^[22]^

The second reason is that the concepts of total blood volume monitoring and fluid responsiveness are often mixed. Studies on IVC diameter are mostly based on the concept of fluid responsiveness.^[19]^ Dehydration describes the loss of total body fluid from intracellular compartments (cut-off value of 5% fluid loss), while hypovolemia describes fluid loss from the intravascular compartment (does not account for total body fluid). Fluid responsiveness is arbitrarily defined as a 10% or greater increase in stroke volume in response to a fluid challenge (intravenous bolus of 500 ml of fluid or passive leg raise), and patients are grouped as either “responders” or “nonresponders.”^[23]^ A patient may be fluid responsive in a hypovolemic (eg, distributive shock), euvolemic, or hypervolemic state, independent of the intravascular fluid state. For this reason, the results obtained from the studies of Orso and Riccardi et al are controversial. Another problematic reason is insufficiency of BUN and Cr values to define dehydration especially in elderly patients.^[3]^ Because the increased BUN/Cr ratio may not be due to only dehydration, it may be caused by other aetiologies such as hemorrhage, renal insufficiency, heart failure (congestive), loss of skeletal muscle with aging, increased protein intake, use of glucocorticoids and so it is problematic to use in the diagnosis of dehydration.^[5]^

This study has some limitations. Since the sonographic IVC measurements are not repeated by a second physician, the operator is exposed to bias. The sample group was relatively small and heterogeneous regarding comorbidities and their effect on the results. Because there is small number of patients admitted to our center for hip fractures. So when we designed this study as a pilot study. The study patients are elderly and may have associated comorbidities such as pulmonary embolism or pulmonary hypertension that were unidentified despite careful examinations. These comorbidities can increase both IVC size and right atrial pressures, which can affect the results. Moreover, lung hyperinflation due to acute exacerbation of asthma or COPD, auto-PEEP and increased intrathoracic pressure may occur. This situation may cause dilatation of IVC at end expirium in preoperatively undiagnosed patients.^[24]^ The delay between hip fracture and the onset time of fluid resuscitation may be another factor affecting the outcome.

## 5 Conclusion

No relationship was found between bedside USG measurement of IVC parameters and BUN/Cr ratios in patients who underwent hip fracture surgery to predict preoperative dehydration. We think that sonographic IVC measurements may be inadequate to predict dehydration in spontaneously breathing elderly patients. As stated in previous large series, it is a more accurate approach to use as an effective tool in determining fluid responsiveness not for intravascular volume assessment. Moreover, BUN/Cr ratio is a poor indicator of severe dehydration and/or occult hypovolemia.

## Author contributions

**Conceptualization:** Ayhan Kaydu.

**Data curation:** Ayhan Kaydu, Erhan Gökçek.

**Formal analysis:** Ayhan Kaydu.

**Funding acquisition:** Erhan Gökçek.

**Investigation:** Ayhan Kaydu, Erhan Gökçek.

**Methodology:** Ayhan Kaydu.

**Project administration:** Ayhan Kaydu.

**Resources:** Ayhan Kaydu, Erhan Gökçek.

**Software:** Erhan Gökçek.

**Supervision:** Ayhan Kaydu.

**Validation:** Ayhan Kaydu.

**Writing – original draft:** Ayhan Kaydu.

**Writing – review and editing:** Ayhan Kaydu.

Ayhan Kaydu orcid: 0000-0002-7781-8885.
